# Barriers to consulting for symptoms of possible colorectal cancer among rural patients in England: A cross‐sectional survey

**DOI:** 10.1111/jrh.70142

**Published:** 2026-03-30

**Authors:** Emily J. Haworth, Linda Sharp, Jennifer Deane, Christina Ellwood, Gregory P. Rubin, Peter Murchie, Sara Macdonald, Lorraine Angell, Christina M. Dobson

**Affiliations:** ^1^ Population Health Sciences Institute Newcastle University Newcastle UK; ^2^ JJR MacLeod Centre for Diabetes Endocrinology and Metabolism Aberdeen Royal Infirmary Aberdeen UK; ^3^ Division of Applied Health Sciences Section of Academic Primary Care University of Aberdeen Aberdeen UK; ^4^ School of Health and Wellbeing University of Glasgow Glasgow UK; ^5^ Independent Researcher Newcastle upon Tyne UK

**Keywords:** colorectal cancer, help‐seeking, inequalities, rural, survey

## Abstract

**Background:**

Rural cancer inequalities are well‐documented globally, but their causes remain unclear. Late diagnosis may impact rural cancer outcomes; however, little is known about rural patients’ beliefs and experiences of consulting for cancer symptoms.

**Methods:**

3400 eligible patients from four primary care practices in rural England were invited to participate. Participants completed a survey about recent symptomatic experience and beliefs about accessing health care. Barriers to accessing primary care were grouped into three domains (individual level, primary care, contextual) and scored. ANOVAs (analysis of variance) were used to investigate variation in scores by fixed socio‐demographic characteristics.

**Results:**

722 surveys were returned (response rate = 21%). Consultation for symptoms was significantly associated with deprivation and age. Participants in the least rural areas, or registered at larger practices, had significantly higher mean scores for barriers to help‐seeking across all three domains. Participants in the least deprived areas had the highest scores across all three domains. Women, younger participants, and those currently working had significantly higher scores for contextual barriers. The most commonly reported barriers to help‐seeking were work commitments, appointment availability, relationship with General Practitioners and road infrastructure.

**Conclusions:**

This is one of the first studies to undertake a granular examination of help‐seeking behaviors and barriers for rural residents. We found no association between degree of rurality and likelihood of consultation. The higher reporting of help‐seeking barriers among people in the least remote areas, or registered at larger practices, challenges assumptions that the most remote patients face the greatest obstacles to consultation.

## INTRODUCTION

Rural cancer inequalities have been well‐documented, globally, for decades, with rural patients facing 5% poorer survival than urban patients.^1^, ^2^, ^3^ The underlying cause, or causes, of these inequalities have yet to be determined, although they appear to be complex.^4^

In the context of cancer, diagnostic delays are associated with more advanced disease at diagnosis and subsequently poorer survival.^5^ There is evidence to suggest that people living in rural areas experience longer diagnostic intervals (time from first presentation to diagnosis) than those in urban areas, which may be contributing to poorer outcomes.^6^, ^7^ For instance, rural lung and colorectal cancer (CRC) patients are more likely to be diagnosed with more advanced disease than urban patients, suggesting something has impeded timely diagnosis for rural patients.^8^, ^9^ In Western Australia, rural patients have longer symptom appraisal periods than those in urban areas, which has been suggested to possibly be the result of rural machismo and stoicism slowing down help‐seeking for symptoms.^10^ Once patients do consult about symptoms, referral to a specialist may take longer, with patients in rural Scotland being significantly more likely to have three or more consultations for cancer symptoms prior to referral, than urban patients.^11^ However, once patients reach specialist assessment and are within the hospital system, the most remote rural patients have equitable, if not shorter, secondary care intervals (time from specialist referral to diagnosis), than urban patients.^12^, ^13^

Distance to health care services has been hypothesised to be an important mediator of rural cancer outcomes, however, evidence relating to this has been mixed. In Denmark, increased distance to primary care was associated with shorter diagnostic intervals, whilst increased distance to specialist cancer diagnostic services was associated with longer diagnostic intervals.^14^ In Scotland increased distance to a specialist cancer center was associated with shorter diagnostic intervals, however, survival was still poorer.^13^ Furthermore, Scottish patients who lived furthest away from primary care centers were more likely to be diagnosed following an emergency presentation, thought to be a result of delayed help‐seeking for symptoms.^15^

There is scant evidence, to date, as to the underlying mechanisms of rural cancer inequalities. The limited previous research that has been undertaken has been conducted in distinct countries with diverse rural environments, infrastructures and health care systems, making generalization to different health care and sociocultural settings challenging.^10^, ^11^, ^14^, ^15^, ^16^ Previous research has also often treated “rural” as the binary opposite of “urban.” Such approaches are problematic as they fail to account for the complexity, heterogeneity and diversity of rural people, communities, infrastructure and economies.^17^ Nuanced approaches to rural cancer inequalities and rural populations are crucial to inform future service (re)design, intervention development, and policy changes, to support earlier presentation and effectively address rural cancer inequalities.

This study was one of the first to take a granular approach to examining mediators of help‐seeking among individuals with potential CRC symptoms in rural England. The qualitative element of this study, which is reported elsewhere, explored people's accounts and experience of barriers to help‐seeking for possible symptoms of CRC, highlighting the impact of seasonal work and self‐employment, and relationships with General Practitioners (GPs), on willingness to consult.^18^ The survey data presented here provides a broader perspective on consultation behavior and potential barriers to help‐seeking among rural residents with symptoms of possible cancer.

## METHODS

### Participant identification and recruitment

Four primary care practices in North Yorkshire, England, served as recruitment sites. Each practice was located in a rural area, which in the United Kingdom is categorised as an area with <10,000 inhabitants.^19^ Rural areas and populations are highly heterogeneous and therefore our sampling strategies sought to ensure that we captured a breadth of characteristics, experiences, and views.^20^ We did this by recruiting from primary care practices which serve diverse rural populations, in relation to population density, local economy, degree of remoteness, as well as varying practice sizes (see Table S1 for practice characteristics).^21^ A general population sample, as opposed to those who had consulted with relevant symptoms, was eligible for the study. This enabled us to examine the experiences and beliefs of those who did—and importantly also those who did not—consult about their symptoms.

At each of the study sites all patients aged 40 years old and over, and able to provide informed consent, were identified by practice staff. A random sample of 850 eligible patients from each site was then generated, using Microsoft Excel, and invited to participate (*n* = 3400 patients across all four practices). Invited patients were sent a study invitation pack through the post, containing an invitation letter, information sheet, and survey. Those who wished to take part completed the survey and consent form and returned it directly to the university research team. Recruitment commenced in November 2019, with practices 1–3 inviting patients consecutively until March 2020. Patients from practice 4 were invited in January 2021, following a pause in recruitment due to the COVID‐19 pandemic.

The survey was adapted from that used in the USEFUL study^22^ and comprised three sections. Section A asked whether the individual had experienced relevant bowel symptoms in the preceding 8 weeks, including diarrhoea, constipation, change in bowel habit, unusual bleeding (in the stool or toilet bowl), or stomach pain. Questions included date of onset; severity of symptom; and whether they had spoken to anyone about their symptom (and whom). It should be noted that not all respondents experienced a symptom.

Section B explored health care access, including distance to primary care (miles and time); distance to hospital (miles and time); and presence of perceived barriers to accessing GP services when unwell (y/n). Participants who reported experiencing barriers to help‐seeking were able to leave free text responses describing the barriers they faced. A series of 18 statements, adapted from the USEFUL study (Table S2), scored using a 5‐point Likert scale (from strongly disagree to strongly agree), examined attitudes toward accessing health care.^22^

Section C collected demographic information, including age; gender; ethnicity; employment; education; and postcode, from which index of multiple deprivation (IMD) score^23^ and rural category^19^ were derived. Questionnaire responses were input into Microsoft Excel and 20% of cases were checked for data accuracy, after which point the final dataset was imported into Stata.

### Data analysis

All surveys, including those partially completed, were included in the analysis, which was done using STATA 18.5.

Numbers and percentages of respondents who reported experiencing one or more relevant symptom(s) were computed, as was the percentage of those who consulted primary care. Tests of association (chi‐squared) were undertaken to examine associations between patient and practice characteristics, and consultation. For the purposes of analysis, GP practices were grouped into “smaller” and “larger” practices, with smaller practices having list sizes <7500 patients, and larger practices categorised as those with ≥7500 patients. Two practices were in each category. Participants were categorised according to deprivation category of the area in which they lived, using the 2019 Index of Multiple Deprivation^23^ and by urban/rural classification.^19^

Likert scale responses to section B statements were scored 1 to 5 (1 = strongly disagree and 5 = strongly agree). Five questions were reverse coded, because of the phrasing of the statement. A high score, therefore, indicated higher barriers to accessing health care services. The research team, including academic, clinical and lay experts, collaboratively agreed statement groupings into three domains: individual barriers, primary care barriers, contextual barriers.

Each domain was analysed separately. Statement scores were summed to produce a total score for each domain; this was then divided by the number of statements in the domain to produce a score between 1 and 5. Pro‐rating, a form of imputation often used to address missing responses within multi‐item scales (see, e.g., EORTC QLQ‐C30 measure of Quality of Life^24^), was applied where a participant had responded to at least 50% of (but not all) statements in a domain; missing responses were replaced by the mean for statements answered. Imputation generally produces less biased estimates than complete case analysis (i.e., restricting analysis to those participants who answered all questions) under a range of missing data scenarios.^25^ Those who responded to less than half of the statements in any domain were excluded from the analysis for that domain. A series of ANOVAs (analysis of variance) were conducted to compare mean domain scores by fixed characteristics (sex, IMD, age, rural category, employment, education, GP practice size, distance and travel time to primary care).^19^, ^23^

Responses to the open‐ended question, “Is there anything that would prevent you from going to your GP, if you felt that you needed to?,” were independently categorised by two researchers, then discussed until consensus was reached on categorisation of responses. Chi‐squared tests were used to examine associations between self‐reporting of barriers and demographic characteristics.

If any participant's data was incomplete, they were excluded from the analysis related to the missing information, but included in all subsequent analysis.

## RESULTS

A total of 722 surveys were returned (21% response rate) and participant characteristics are presented in Table 1 (see Table S3 for participant characteristics by practice).

**TABLE 1:** Participant characteristics.

Demographic characteristic	*n* (%)
Gender	
Male	372 (52%)
Female	324 (45%)
*Missing*	26 (4%)
Age	
<60	183 (25%)
60–64	126 (17%)
65–74	235 (33%)
≥75	144 (20%)
*Missing*	34 (5%)
Ethnicity	
White—British	665 (92%)
White—Other	2 (0%)
Asian	2 (0%)
*Missing*	53 (7%)
Education	
Degree or postgraduate degree	238 (33%)
Professional qualifications	211 (29%)
High school qualifications	159 (22%)
No educational qualifications	73 (10%)
*Missing*	41 (6%)
Employment	
Employed	247 (34%)
Retired	421 (58%)
Unemployed	16 (2%)
Full time parent/carer	10 (1%)
Student	1 (0%)
*Missing*	27 (4%)
Rural category	
Rural town and fringe (category D)	208 (29%)
Rural village (category E)	454 (63%)
Rural hamlet and isolated dwelling (category F)	17 (2%)
*Missing*	43 (6%)
IMD	
4, 5, 6 (more deprived)	398 (55%)
7, 8	91 (13%)
9, 10 (least deprived)	196 (27%)
*Missing*	37 (5%)
Practice size	
Larger practices	317 (44%)
Smaller practices	405 (56%)
*Missing*	0
Distance to primary care	
Under a mile	293 (41%)
1–5 miles	285 (39%)
Over 5 miles	141 (20%)
*Missing*	3 (0.42%)
Travel time to primary care	
Under 15 min	542 (75%)
Over 15 min	177 (24.51%)
*Missing*	3 (0.42%)

### Help‐seeking for symptoms

A total of 267 respondents (37%) stated that they had experienced a relevant symptom in the 8 weeks preceding the survey (see Table S4). Of those who experienced symptoms, 48% of respondents experienced a single symptom (*n* = 128), 29% reported 2 symptoms (*n* = 78), 15% reported 3 (*n* = 40), 7% reported 4 (*n* = 18), and 2% reported 5 symptoms (*n* = 5). Overall, 34% of symptomatic survey respondents consulted primary care about their symptoms (*n* = 92).

Likelihood of consultation varied based on several factors. The number of symptoms experienced was significantly associated with likelihood of consultation (χ^2^(3) = 10.15, *p* = 0.02). Only 27% of those who reported one symptom consulted primary care, compared to 35% of respondents experiencing two symptoms, 38% of those experiencing three symptoms and 62% of those who reported experiencing four or more.

Participants were most likely to consult if they experienced bleeding (*n* = 18, 42%), followed by a change in bowel habits (*n* = 23, 37%), diarrhoea (*n* = 32, 29%), stomach pain (*n* = 40, 29%), and constipation (*n* = 33, 24%).

Consultation for symptoms also varied by demographic characteristics (see Table 2). IMD was significantly associated with consultation, with those living in less deprived areas more likely to consult about their symptoms. Older participants were also significantly more likely to consult (i.e., greater than 74 years old, 58%; less than 60 years old, 23%).

**TABLE 2:** Associations between consultation for symptoms and demographic characteristics.

Demographic characteristic	*n* (%) consulted	*p* value
Sex		0.493
Female	52 (33%)	
Male	37 (37%)	
IMD		0.029
4, 5, 6 (more deprived)	44 (30%)	
7, 8	10 (26%)	
9, 10 (least deprived)	35 (46%)	
Rural category		0.497
D (Rural town)	30 (39%)	
E (Rural village)	53 (32%)	
F (Rural hamlet)	6 (35%)	
Employment		0.119
Working	26 (28%)	
Not working	63 (38%)	
Education		0.825
Degree or postgraduate degree	28 (31%)	
Professional qualifications	30 (36%)	
High school qualifications	22 (32%)	
No educational qualifications	8 (40%)	
Age		<0.001
<60	17 (23%)	
60–64	15 (34%)	
65–74	25 (28%)	
>74	30 (58%)	
Practice size		0.853
Larger practices	44 (35%)	
Smaller practices	47 (34%)	
Distance to primary care		0.112
Under a mile	43 (41%)	
1–5 miles	35 (30%)	
Over 5 miles	12 (27%)	
Travel time to primary care		0.312
Under 15 min	72 (35%)	
Over 15 min	18 (29%)	

### Barriers to help‐seeking

All 722 participants provided at least partial responses to the perceived barriers to accessing health care services questions (see Table 3). After those who did not answer the required number of questions for the domain were excluded, there were a total of 688 responses within the individual barriers domain, 691 responses within the primary care barriers domain and 689 responses in the contextual barriers domain. Overall, the highest mean score for barriers reported was within the individual barriers domain, and the lowest mean score for barriers to help‐seeking reported was in the domain of contextual barriers (Table 3).

**TABLE 3:** Associations between perceived barriers to accessing health care services and demographic characteristics mean domain scores, standard deviations and *p* values for ANOVA tests.

	Domain					
	Individual barriers		Primary care barriers		Contextual barriers	
Demographic characteristic	Mean (SD)	*p* value	Mean (SD)	*p* value	Mean (SD)	*p* value
Overall	2.85 (0.03)	–	1.96 (0.03)	–	1.56 (0.03)	–
Sex						
Female	2.88 (0.90)	0.424	2.02 (0.69)	0.008*	1.61 (0.68)	0.046
Male	2.82 (0.90)		1.89 (0.62)		1.51 (0.63)	
IMD						
4, 5, 6 (more deprived)	2.74 (0.87)	<0.001*	1.84 (0.64)	<0.001*	1.44 (0.64)	<0.001*
7, 8	3.10 (0.96)		2.15 (0.67)		1.76 (0.72)	
9, 10 (least deprived)	2.97 (0.92)		2.12 (0.64)		1.69 (0.62)	
Age						
<60	2.91 (0.87)	0.557	2.07 (0.70)	0.081	1.72 (0.72)	<0.001*
60–64	2.78 (0.88)		1.96 (0.67)		1.52 (0.57)	
65–74	2.81 (0.89)		1.93 (0.65)		1.46 (0.62)	
>74	2.89 (0.97)		1.89 (0.61)		1.55 (0.69)	
Rural category						
Rural town and fringe (category D)	3.08 (0.99)	<0.001*	2.20 (0.67)	<0.001*	1.84 (0.64)	<0.001*
Rural village (category E)	2.76 (0.89)		1.85 (0.63)		1.42 (0.62)	
Rural hamlet and isolated dwelling (category F)	2.73 (0.96)		2.14 (0.72)		1.63 (0.70)	
Employment						
Working	2.89 (0.86)	0.351	1.99 (0.65)	0.455	1.71 (0.72)	<0.001*
Not working	2.83 (0.92)		1.95 (0.66)		1.48 (0.61)	
Education						
Degree or postgraduate degree	2.80 (0.84)	0.201	2.04 (0.72)	0.122	1.54 (0.72)	0.666
Professional qualifications	2.88 (0.89)		1.89 (0.61)		1.59 (0.64)	
High school qualifications	2.85 (0.92)		1.95 (0.60)		1.61 (0.62)	
No educational qualifications	3.07 (1.07)		1.99 (0.72)		1.52 (0.62)	
Practice size						
Larger practices	3.11 (0.93)	<0.001*	2.20 (0.63)	<0.001*	1.82 (0.68)	<0.001*
Smaller practices	2.65 (0.82)		1.78 (0.62)		1.36 (0.57)	
Distance to primary care						
Under a mile	2.96 (0.93)	0.031*	2.01 (0.65)	0.018*	1.61 (0.67)	0.103
1–5 miles	2.77 (0.85)		1.98 (0.67)		1.56 (0.67)	
Over 5 miles	2.79 (0.91)		1.82 (0.64)		1.47 (0.62)	
Time to primary care						
Under 15 min	2.85 (0.89)	0.835	2.00 (0.66)	0.025*	1.58 (0.66)	0.220
Over 15 min	2.84 (0.91)		1.87 (0.64)		1.51 (0.65)	

* *p* value <0.05.

### Individual barriers

Rural category and IMD were both significantly associated with individual barriers domain scores. The least remote respondents (category D) had higher mean scores for individual barriers than those in more remote areas (mean barrier score = 2.73 for category F; 2.76 for category E; and 3.08 for category D). Participants in the less deprived areas also had a significantly higher mean score for reported individual barriers (mean score = 3.10) compared to participants residing in more deprived areas (mean barrier score = 2.74). Patients at larger GP practices scored significantly higher in individual barriers (mean = 3.11) to accessing primary care, than those at smaller GP practices (mean = 2.65). Those living 1–5 miles from primary care had significantly lower mean scores for reported individual barriers (mean = 2.77) than those over 5 miles (mean = 2.79) and those under a mile (mean = 2.96). There was no significant association between sex, employment, education or travel time and reported individual barriers.

### Primary care barriers

Mean scores in the primary care barriers domain were significantly associated with rural category, with participants in the least rural area having the highest mean score (category D mean barrier score = 2.20; category E mean score = 1.85; and category F mean score = 2.14). IMD was significantly associated with reported primary care barriers: those residing in the least deprived areas had a higher mean barrier score of 2.12, compared to a mean score of 1.84 in more deprived areas. Females also had significantly higher mean scores for primary care barriers (mean score = 2.02), compared to males (mean score = 1.89). Those at larger GP practices scored significantly higher (mean = 2.20) than those at the smaller GP practices (mean = 1.78). Those living over 5 miles from primary care had significantly lower mean scores for primary care barriers (mean = 1.82), than those living under a mile away (mean = 2.01). Those who had a travel time of over 15 minutes to primary care scored significantly lower (mean = 1.87) than those who had a travel time of under 15 minutes (mean = 2.00). There were no significant associations between primary care barriers and age, education, employment, or distance.

### Contextual barriers

Rural category, IMD, sex, age, and employment were all significantly associated with mean contextual barriers domain scores. Those who scored higher in this domain were: the least remote respondents (category D mean score = 1.84; category E mean score = 1.42; category F mean score = 1.63), those from the less deprived areas (least deprived areas mean score = 1.69, vs. mean score of 1.44 in more deprived areas), females (mean score = 1.61 vs. males mean score = 1.51), participants who worked (mean score for those working = 1.71, compared to a mean score of 1.48 for those not working), and those aged younger than 60 (mean score = 1.72 vs. 1.52 (60–64), 1.46 (64–74), and 1.55 (75+)). Respondents registered at larger GP practices also scored significantly higher for contextual barriers (mean score = 1.82 vs. mean score of 1.36 for reported contextual barriers at smaller GP practices). There were no significant associations between contextual barriers and education, distance, or travel time.

### Self‐reported barriers to help‐seeking

Seventy‐six respondents reported experiencing barriers to help‐seeking (see Table 4 for associations between reporting of barriers and participant characteristics). Analyses showed that women, those who were employed, and younger participants were significantly more likely to report barriers.

**TABLE 4:** Associations between self‐reported barriers to help‐seeking and demographic characteristics.

Demographic characteristic	*n* (%) reported barriers	*p* value
Sex		
Female	49 (13%)	0.030
Male	26 (8%)	
IMD		
4, 5, 6 (more deprived)	36 (9%)	0.356
7, 8	11 (12%)	
9, 10 (least deprived)	25 (13%)	
Rural category		
Rural town and fringe (category D)	26 (13%)	^a^
Rural village (category E)	43 (10%)	
Rural hamlet and isolated dwelling (category F)	2 (12%)	
Employment		
Working	38 (15%)	0.004
Not working	37 (8%)	
Education		
Degree or postgraduate degree	26 (11%)	0.341
Professional qualifications	29 (14%)	
High school qualifications	15 (9%)	
No educational qualifications	5 (7%)	
Age		
>60	36 (20%)	<0.001
60–64	13 (10%)	
65–74	17 (7%)	
>74	8 (6%)	
Practice size		
Larger practices	35 (11%)	0.675
Smaller practices	41 (10%)	
Distance to primary care		
Under a mile	31 (11%)	0.574
1–5 miles	27 (10%)	
Over 5 miles	18 (13%)	
Time to primary care		
Under 15 min	52 (10%)	0.132
Over 15 min	24 (14%)	

^a^ Numbers in cells are too small for test result to be robust.

**FIGURE 1:**
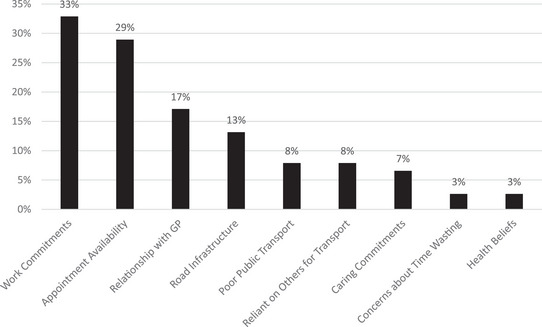
Reported barriers to help‐seeking.

All 76 respondents provided free‐text responses about perceived barriers to accessing primary care. Some reported multiple barriers, resulting in a total of 91 barriers that were categorised (see Figure 1; example comments for each category are presented in Table S5).

## DISCUSSION

Analyses found no significant association between likelihood of consultation and rural category of residence. However, respondents residing in the least rural areas, or registered at larger practices, did report significantly more barriers to consultation. Reported barriers included systemic, structural, and practical issues (i.e., health care systems, infrastructure, remoteness, and landscape) that shaped help‐seeking decision‐making, in addition to distance and travel time. These reported barriers triangulate with interview findings from this study, whereby patients registered at larger practices were often reluctant to consult, and/or re‐consult about symptoms. Belief that the GP wouldn't help or perceived difficulty around talking to new GPs who they felt did not know them or their medical and social history were both frequently raised.^18^

Patients' perceptions of their relationship with their GP have been shown to be better at smaller primary care practices, with patients at smaller practices also feeling that they have better access to primary care practitioners.^26^, ^27^, ^28^ Other work has shown that rural patients would rather wait longer for an appointment with their named doctor, than be seen sooner by one they did not know. Practices that schedule patient appointments with the first available doctor, as opposed to scheduling appointments with a named GP, also have lower levels of patient satisfaction, as patients report experiencing difficulty establishing relationships and building rapport with new doctors.^29^, ^30^

Survey findings relating to deprivation were complex. Participants in more deprived areas were less likely to consult about their symptoms, a finding which aligns with existing evidence showing that increased deprivation is associated with longer patient intervals, increased likelihood of presentation through emergency routes, and more advanced disease at diagnosis.^31^, ^32^ Deprivation has been shown to be associated with cancer symptom awareness and recognition, with individuals in the most deprived areas less likely to consult about symptoms.^33^ However, our analyses showed that respondents residing in the *least* deprived areas were significantly more likely to report barriers to help‐seeking.

Health literacy describes the ability of individuals to find, understand and use information and services to make informed health‐related decisions, including interpreting health information and navigating the health care system. Health literacy is a known barrier to accessing health care and also follows a socio‐economic gradient.^34^, ^35^ It may be that higher health literacy in the least deprived areas contributes to higher reporting of barriers to help‐seeking, as these patients can more readily identify and articulate perceived barriers to presentation, as well as having higher expectations of the health care system.

The parallel between higher reporting of barriers in the least rural, and least deprived areas, despite this not mirroring likelihood of consultation, is of particular interest. In England, the least rural areas (rural towns and fringes) have a notably higher proportion of individuals of higher socio‐economic status (SES).^36^ It may be, therefore, that the higher reporting of barriers in the least rural areas is due, at least in part, to SES and reflects the association between socio‐demographic characteristics and health literacy. The link between rural cancer inequalities and deprivation is evident internationally, including in countries such as the United States and Australia.^37^, ^38^ It has been suggested that the inter‐relationships between deprivation, remoteness and likelihood of consultation in these countries may be driven by financial issues present in insurance‐based health care systems. However, the fact that similar findings have arisen in this work—undertaken in a publicly‐funded English health care system where care is free at the point of delivery—suggests that the intersection between remoteness, deprivation and help‐seeking extends beyond insurance‐based access, and may include the finanical impact of travel and hotel costs for rural patients, arising from lack of local oncology services in rural areas^39^ as well health literacy, and cultures of stoicism.^18^

Older participants living in rural areas were more likely to consult when experiencing symptoms of possible bowel cancer. Previous research examining the influence of age and help‐seeking in the general population has been mixed, with some showing older people being more likely to take longer to present whilst others found the opposite.^31^, ^40^ However, these studies do not consider the influence of rurality on the likelihood of consultation across different age‐groups, and how pressures unique to rural populations (i.e., lack of public transport, self‐employment, or diminishing services) may intersect with timeliness of consultation. Women, younger participants, and those who were working, were more likely to report higher contextual barriers, unsurprising given that competing priorities have a substantial impact on help‐seeking decision‐making generally, as well as in rural populations specifically.^41^, ^42^, ^43^, ^44^ This is supported by the finding that work commitments were the most reported barrier to help‐seeking by survey respondents, which, again, is particularly pertinent in rural areas given high levels of seasonal work and self‐employment across agriculture and tourism in these areas.^45^, ^46^

### Strengths and limitations

To our knowledge, this is one the first studies to take a granular approach to examining rural patients’ engagement with primary care, as opposed to looking at rural and urban as dichotomous categories. Within this study, we did not investigate patient interval length, analysis of which would have allowed further examination of rural help‐seeking decision‐making and timeliness. Future research taking a nuanced approach to examination of patient interval length within and across rural populations is needed to better understand the factors contributing to rural cancer inequalities.

A limitation of this study is the response rate of 21%. There is the possibility that the respondents recruited to the study are not representative of the wider population, meaning that the results are not generalisable. Of the sample recruited, however, respondents were representative in relation to both overall demographic characteristics and the prevalence of reported symptoms. Of the 722 respondents, 37% reported experiencing symptoms in the preceding 8 weeks, which aligns with the anticipated prevalence of gastrointestinal symptoms in the general population, of 30%.^47^ Our response rate is also similar to other studies exploring cancer symptom experience and behavior in both the general population and those who had presented to primary care about symptoms.^22^, ^48^

In this study, patients were recruited from one region of England. Given the heterogeneity of rural communities, it may be that the findings do not directly translate to other rural populations in England, or internationally. Further work, and international comparison, is warranted to understand how these findings translate to other rural communities, however, the parallels in our findings and evidence from rural research globally suggest that these findings may be pertinent across health care systems and rural communities.

The grouping of statements within domains was undertaken through an iterative decision‐making process within the study team, comprising lay, clinical, and academic experts. This could be viewed as a limitation and another approach—such as factor analysis—may have been more objective.

Finally, although there was a substantial sample size, certain categories had a limited number of respondents preventing us from exploring associations. However, the majority of participants were from the more rural areas, for whom the study findings are most salient.

## CONCLUSION

It has been suggested that rural cancer inequalities are driven by distance to health care services; however, our findings challenge this. We have presented a granular examination of likelihood of, and barriers to, consultation for possible symptoms of CRC with a large rural patient population. Participants residing in the least rural areas, and those registered at larger practices reported more barriers to help‐seeking than those registered at smaller practices, or those residing in more rural/remote areas. The complex relationship between rurality, SES and help‐seeking behaviors and attitudes requires further examination internationally, to understand the impact of, and relationship between different health systems and service provision, rurality and help‐seeking, to enable effective intervention addressing rural inequalities to be implemented.

This analysis highlights the need for further research to identify modifiable factors that improve likelihood of consultation for symptoms of possible cancer among rural patients. Mechanisms to facilitate consultation during periods of seasonal pressure in rural environments (i.e., harvest, lambing or summer tourism), such as weekly drop‐in clinics, may be particularly beneficial and pilots of these in rural Northern England are proving effective.^49^ These findings also highlight the importance of further examination into patient interval length among patients residing in areas of differing degrees of rurality and different practice sizes to understand help‐seeking behavior and identify populations who may be most vulnerable to diagnostic delays. Addressing barriers to presentation in rural populations has substantial potential to increase early diagnosis, improve survival, and reduce rural cancer inequalities.

## CONFLICT OF INTEREST STATEMENT

The authors declare no competing interests.

## STATEMENT OF CONSENT

All participants provided written, informed consent, which they returned to the study team alongside their completed questionnaire.

## ETHICS STATEMENT

Ethical approval for this study was obtained from the NHS National Research Ethics Service (NRES) Wales REC 1 Committee (REC Ref: 19/WA/0198) and research was undertaken in accordance with the principles of The Declaration of Helsinki (2024).

## Supporting information



Supporting Information

## Data Availability

The datasets generated during the current study are not publicly available as participants did not provide consent for access to their anonymised data for the purposes of future research.
