# Supplementary material for: Barriers to consulting for symptoms of possible colorectal cancer among rural patients in England: A cross‐sectional survey
Source: J Rural Health. 2026 Mar 30;42(2):e70142. doi: 10.1111/jrh.70142 (PMC13034810; doi:10.1111/jrh.70142)
Supplement: Supplementary file 1 — Supporting Information [file JRH-42-0-s001.docx]

# Supplementary Material

*Supplementary table 1. Practice characteristics*

| **Practice characteristic** | **Practice 1** | **Practice 2** | **Practice 3** | **Practice 4** |
| --- | --- | --- | --- | --- |
| **List size** | 7,343 patients | 10,457 patients | 1,185 patients | 1,547 patients |
| **Number of GPs** | 7 | 9 | 2 | 1 |
| **Location** | Rural town and fringe | Rural town and fringe in sparse setting | Rural village and dispersed | Rural village and dispersed in a sparse setting |
| **N participants recruited** | 178 | 139 | 125 | 280 |

*Supplementary Table 2: Barrier Domains and Statements*

| **Individual barriers** | **Primary Care barriers** | **Contextual barriers** |
| --- | --- | --- |
| It is a waste of the GP’s time to see patients with symptoms that might not be serious | I would feel confident discussing any type of symptom with the GP | It is difficult for me to arrange transport to the GP’s surgery |
| I would only go to the GP once I was no longer able to tolerate my symptoms | I feel embarrassed talking to the GP about my illnesses | I am too busy to make time to visit the GP |
| Other people go to the GP a lot more than I do | I worry that the GP would think that I am foolish if I went to the doctor’s for something that turned out to be minor | It is difficult to make at an appointment with the GP |
| Visiting the GP with symptoms that may not be serious is a waste of my time | I find it easy to ask the GP for the care or treatment that I think I need | It is easier to go to A&E when I’m ill, than it is to go to see the GP |
| I have lot of things (other than my health) to worry about | The GP gives me the opportunity to ask questions | It is easier to go to a pharmacy when I’m ill, than it is to see the GP |
|  | The GP encourages me to mention all my health concerns during the consultation |  |
|  | The GP is difficult to talk to |  |
|  | I have built a relationship with the GP and feel that they know me |  |

*Supplementary table 3. Demographic characteristics of participants at each practice*

| **Demographic characteristic** | | **Practice 1 n(%)** | **Practice 2 n(%)** | **Practice 3 n(%)** | **Practice 4 n(%)** | **Overall n(%)** |
| --- | --- | --- | --- | --- | --- | --- |
| **Gender** | Male | 73 (41%) | 69 (50%) | 54 (43%) | 128 (46%) | 372 (52%) |
|  | Female | 97 (54%) | 67 (48%) | 67 (54%) | 141 (50%) | 324 (45%) |
|  | Missing | 8 (4%) | 3 (2%) | 4 %3%) | 11 (4%) | 26 (4%) |
| **Age** | <60 | 47 (26%) | 33 (24%) | 32 (26%) | 71 (25%) | 183 (25%) |
|  | 60-64 | 23 (13%) | 27 (19%) | 30 (24%) | 46 (16%) | 126 (17%) |
|  | 65-74 | 60 (34%) | 44 (32%) | 38 (30%) | 93 (33%) | 235 (33%) |
|  | ≥75 | 35 (20%) | 30 (22%) | 20 (16%) | 59 (21%) | 144 (20%) |
|  | *Missing* | 13 (7%) | 5 (4%) | 5 (4%) | 11 (4%) | 34 (5%) |
| **Ethnicity** | White- British | 161 (90%) | 131 (94%) | 116 (93%) | 257 (92%) | 665 (92%) |
|  | White – Other | 1 (1%) | 1 (1%) | 0 | 0 | 2 (0%) |
|  | Asian | 0 | 1 (1%) | 1 (1%) | 0 | 2 (0%) |
|  | *Missing* | 16 (9%) | 6 (4%) | 8 (6%) | 23 (8%) | 53 (7%) |
| **Education** | Degree or postgraduate degree | 55 (31%) | 39 (28%) | 41 (33%) | 103 (37%) | 238 (33%) |
|  | Professional qualifications | 52 (29%) | 47 (34%) | 39 (31%) | 73 (26%) | 211 (29%) |
|  | High school qualifications | 46 (26%) | 31 (22%) | 29 (23%) | 53 (19%) | 159 (22%) |
|  | No educational qualifications | 19 (11%) | 11 (8%) | 14 (11%) | 29 (10%) | 73 (10%) |
|  | *Missing* | 6 (3%) | 11 (8%) | 2 (2%) | 22 (8%) | 41 (6%) |
| **Employment** | Employed | 57 (32%) | 44 (32%) | 49 (39%) | 97 (35%) | 247 (34%) |
|  | Retired | 107 (60%) | 84 (60%) | 66 (53%) | 164 (59%) | 421 (58%) |
|  | Unemployed | 4 (2%) | 3 (2%) | 4 (3%) | 5 (2%) | 16 (2%) |
|  | Full time parent/carer | 1 (0.56%) | 4 (3%) | 2 (2%) | 3 (1%) | 10 (1%) |
|  | Student | 1 (0.56%) | 0 | 0 | 0 | 1 (0%) |
|  | *Missing* | 8 (4%) | 4 (3%) | 4 (3%) | 11 (4%) | 27 (4%) |
| **Rural category** | Rural town and fringe (category D) | 96 (54%) | 98 (71%) | 0 | 14 (5%) | 208 (29%) |
|  | Rural village (category E) | 66 (37%) | 35 (25%) | 104 (83%) | 249 (89%) | 454 (63%) |
|  | Rural hamlet and isolated dwelling (category F) | 5 (3%) | 0 | 12 (10%) | 0 | 17 (2%) |
|  | *Missing* | 11 (6%) | 6 (4%) | 9 (7%) | 17 (6%) | 43 (6%) |
| **IMD** | 4, 5, 6 (more deprived) | 48 (27%) | 57 (41%) | 45 (36%) | 248 (89%) | 398 (55%) |
|  | 7, 8 | 48 (27%) | 16 (12%) | 23 (18%) | 4 (1%) | 91 (13%) |
|  | 9, 10 (least deprived) | 72 (40%) | 60 (43%) | 53 (42%) | 11 (4%) | 196 (27%) |
|  | *Missing* | 10 (6%) | 6 (4%) | 4 (3%) | 17 (6%) | 37 (5%) |
| **Distance to primary care** | Under a mile | 88 (49%) | 73 (53%) | 38 (30%) | 94 (34%) | 293 (41%) |
|  | 1-5 miles | 70 (39%) | 49 (35%) | 58 (46%) | 108 (39%) | 285 (39%) |
|  | Over 5 miles | 20 (11%) | 16 (12%) | 29 (23%) | 76 (27%) | 141 (20%) |
|  | *Missing* | 0 | 1 (1%) | 0 | 2 (1%) | 3 (0.42%) |
| **Travel time to primary care** | Under 15 minutes | 147 (83%) | 113 (81%) | 98 (78%) | 184 (66%) | 542 (75%) |
|  | Over 15 minutes | 31 (17%) | 25 (18%) | 27 (22%) | 94 (34%) | 177 (25%) |
|  | *Missing* | 0 | 1 (0.72%) | 0 | 2 (0.71%) | 3 (0.42%) |
| **Total participants in analysis** | | 178 | 139 | 125 | 280 | 722 |

*Supplementary Table 4. Number (%) of patients reporting each symptom of possible bowel cancer*

| **Symptom** | **N(%)** |
| --- | --- |
| Diarrhoea | 111(15%) |
| Constipation | 136(29%) |
| Change in bowel habits | 62(9%) |
| Bleeding (in stools or toilet bowl) | 43(6%) |
| Stomach pain | 139(20%) |

*Supplementary Table 5. Free text responses categorised into self-reported health-seeking barriers*

| **Self-reported barrier** | **Comment 1** | **Comment 2** | **Comment 3** |
| --- | --- | --- | --- |
| **Work commitments** | historically work used to make it extremely difficult to see my GP - my absence would affect many people and no-one else could do my job Participant NO  Fitting in with work. I do not work full time, so sometimes wait until a non-work day. |  | I'm self-employed and sometimes just too busy. |
| **Appointment availability** | very long waiting time to see a GP - only usually can see nurse practitioner | Difficulty in getting an appointment, feel things are likely to have resolved before appointment date, ours 2 weeks from asking for appointment | Frequently unable to get an appointment inside 3 weeks, receptionist makes people see nurse practitioner. Not sure if there actually is any GP's at my surgery. |
| **Relationship with GP** | lack of confidence in GPs | dislike of doctors |  |
| **Road infrastructure** | if car broke down/icy roads (not on bus route from my village) | road conditions e.g. snow |  |
| **Poor public transport** | Difficulty in getting to hospital/outpatients clinics etc. (very poor public transport links) make me less likely to go. But the actual GPs services are outstandingly good! | If I was unable to drive for any reason (illness, car broken down, snowy weather) as we have no bus service |  |
| **Reliant on others for transport** | getting someone to take me | can't walk without support, can't do smallest steps. Daughter need to take time off work, she works 25 miles away. |  |
| **Caring commitments** | I am my wife’s carer, if she was ill I would postpone my visit until nearest opportunity | work and caring responsibilities - unless any symptoms were extremely bad |  |
| **Concerns about time wasting** | not wanting to waste their time | they are always so busy - I feel a bit of a nuisance. |  |
| **Health benefits** | very anxious, as I am scared of finding out anything is wrong with me | so many things go away if one waits to see |  |
